# Implementation and Evaluation of a School-Based Educational Program Targeting Healthy Diet and Exercise (DIEX) for Greek High School Students

**DOI:** 10.3390/sports10120196

**Published:** 2022-12-01

**Authors:** Maria Angeli, Mary Hassandra, Charalampos Krommidas, Athanasios Kolovelonis, Vassilios Bouglas, Yannis Theodorakis

**Affiliations:** Department of Physical Education and Sport Science, University of Thessaly, 42100 Trikala, Greece

**Keywords:** adolescents, healthy diet, planned behavior theory, health education intervention, life skills, behavior change, exercise

## Abstract

The present study examined the effectiveness of a school-based health education program promoting healthy diet and exercise, named DIEX, implemented on adolescents. The program is based on the Theory of Planned Behavior (TPB) and is supported by life skills training. The recipients of the program were high school students (*n* = 367; 168 boys and 199 girls) from 14 schools in Greece who attended 10 1 h sessions implemented by their schoolteachers. Data were collected before and after the program implementation through online questionnaires for: a. attitude, intention, subjective norms, and PBC toward healthy eating; b. knowledge about healthy eating; c. exercise behavior and attitude toward the DIEX program; and d. satisfaction with the DIEX program (only post-program). The results showed that there were significant differences among the pre- and post-measures of students’ knowledge and behavior about healthy diet, as well as attitudes and satisfaction toward the program’s application. The results show that the DIEX program may have improved nutrition behavior variables. Conclusively, a theory-based behavioral intervention with skills training may result in positive behavioral changes for young students during health education in school settings with the assistance of new technologies.

## 1. Introduction

The Global Nutrition Report [1] highlights the importance of taking care of malnutrition and poor diets, as obesity and nutrition-related disease rates are increasing alarmingly, with no signs of slowing. According to the WHO Europe [2], almost one in eight children between the age of 7 and 8 years old is obese. Overweight children are at elevated risk of becoming obese adults [3]. Data analyses indicate the prevalence of obesity and overweight children and youth globally [4]. The prospects are worsening, as it is estimated that 254 million children and adolescents will be classified as obese by 2030 [5]. On top of that, several studies have stated that overweight youth face multiple health conditions, such as depression, low self-esteem, behavior problems, and bullying [6,7]. Overweight children and adolescents have negative body image and body dissatisfaction [8]. Moreover, obesity is associated not only with mental and psychological issues, but also with serious health problems, such as cardiovascular disease, type 2 diabetes, musculoskeletal disorder, and some forms of cancer [9].

Adolescent health is overlooked in global public health because this age group is regarded as healthy [10]. Healthy diet guidance should be taught to children from early childhood, as those early childhood years are critical for forming healthy eating habits in the long term [11]. Indeed, health risk behaviors are formed during childhood and adolescence [12,13], and school has been recognized as a proper environment for initiating a health education program [14]. The literature suggests that school healthy diet education interventions present promising results in improving adolescents’ healthy diet choices [15].

Reviews have demonstrated that the most efficient health interventions will be those that include a behavioral theory framework [16,17,18]. These theoretical frameworks aim to understand the predictors of human behavior and create a healthy social and physical environment for healthier options [19]. Human behavior is affected by several factors; thus, it is crucial to design a health education intervention by targeting the competence, motivation, and opportunities (social and environmental) that affect the healthy diet of school children. The Theory of Planned Behavior [20] has been proven to be an effective approach for transforming an undesirable behavior into a healthy behavior [21]. It has been used in research on health-related behaviors [22,23], as well as for the evaluation and planning of several behavioral change interventions [24]. According to previous findings, behavioral change interventions would benefit from the use of the TPB framework, as it has been found useful in improving dietary behaviors among youth. Riebl et al. [25] reviewed three interventions, and their findings indicated changes toward healthy eating behavior and modified intentions toward healthy diet choices. More specifically, attitude has been found to be the stronger predictor of intention, and intention is the most significant construct of TPB to predict healthy diet behavior.

According to research, participation in regular exercise is highly recommended to prevent nutrition-related health problems [26]. For example, Drenowatz et al. [27] demonstrated that healthy dietary choices and involvement in sports are related to motor competence, which indicates that they are important contributors to a healthy and active lifestyle [28]. Moreover, Meng et al. [29] evaluated the effectiveness of a two-year obesity prevention program, targeting sports dietary education and healthy eating behaviors, in promoting daily PA and learning life skills among adolescent athletes. The intervention significantly decreased unhealthy food consumption and highlighted the importance of engaging in PA.

A multicomponent approach is needed to apply an effective intervention that aims to prevent childhood obesity, targeting the modification of exercise, lifestyle, and dietary behavior [30]. Thus, testing the validity of a multi-strategy school-based education program targeting the promotion of healthy diet and exercise can contribute to the improvement of existing areas of relevant programs and add knowledge that can be used in the development of future ones.

### 1.1. Theory of Planned Behavior (TPB) and School-Based Educational Programs Targeting Healthy Diets

Relevant reviews have stressed that school-based programs covering a variety of strategies can be effective in promoting healthy diet among youth [31]. Steinmetz et al. [32] reviewed a range of interventions and concluded that the TPB is a popular model for explaining the mechanisms underlying the adoption of healthy behaviors. The TPB, which evolved from a prior theoretical framework, the Theory of Reasoned Action (TRA) [33,34], is an appropriate theoretical framework for considering multiple behavioral changes, as it allows the identification of beliefs related to the production of health-related behaviors within a specific context [35]. According to the TRA, the strongest predictor of behavior is behavioral intention, which indicates whether a person is ready to perform the aforementioned behavior. This behavior, subsequently, is predicted by attitude toward the behavior (the degree to which an individual evaluates the behavior and its outcome as positive or negative) and subjective norms (individual’s comprehension of the social pressure of significant others to participate) [36]. The TPB extends the TRA, with the addition of a third construct, the perceived behavioral control (perceived ability of an individual to control the behavior), which is thought to have a direct impact on the behavior [35].

School-based educational interventions guided by TPB have exhibited a post-intervention positive change in participants’ intentions and dietary behaviors, and they have also been found to improve children’s and adolescents’ behaviors toward healthy diet, increased fruit and vegetable consumption, and decreased junk food consumption [37,38,39], attitude, and perceived behavioral control [39,40]. More specifically, in a recent study by Kaveh et al. [40], female adolescents followed a nutrition education intervention based on the TPB model with the addition of a parental control construct. The results showed increased knowledge after the program’s implementation and an increase for all TPB constructs, indicating the effectiveness of such interventions. According to these findings, Dhauvadel et al. [41] implemented and evaluated a school educational healthy diet intervention among school-going adolescents. Intention and attitude toward healthy eating significantly increased post-test. Similarly, in the study by Harrington et al. [38], they tested the effectiveness of a Summer Food Service Program (SFSP) on high school students’ intentions to improve their eating habits by consuming more fruits and vegetables.

In a recent review, Salam et al. [42] suggested that an effective intervention targeting obesity prevention among adolescents could consist of a combination of behavioral therapy, exercise, and diet. Furthermore, computer-based health education programs are considered a promising strategy [43]. A growing body of evidence suggests that effective whole-school interventions should encompass theory-based approaches, PA, the use of digital technology, and persuasive communication [31,44,45].

School health education programs integrate educational components, which aim to improve students’ well-being [46,47]. In most European countries, health education does not consist of an autonomous project; rather, it constitutes a part of other school courses [48]. Greek health education programs are limited and implemented on a voluntary base by teachers, and they are not included in the compulsory curriculum [49]. Taking these into consideration, it is advisable to develop school-based health education programs to prevent unhealthy behaviors and educate adolescents about the harmful effects of making unhealthy choices.

### 1.2. TPB and School-Based Educational Programs Targeting Exercise

Unhealthy diet in coexistence with low levels of exercise has been shown to have high impact on obesity in youth [50]. Intervention programs based on the TPB framework have been found to be effective in promoting exercise [51]. It has been proposed that such interventions are more effective than other theory-based interventions [52]. Moreover, a combination of exercise and behavior-change strategies has been found to be more effective in maintaining and increasing PA [18].

McIntosh et al. [53] reviewed web-based PA interventions among youth, proposing that theory-based and web-based interventions have been successful at increasing adolescents’ PA levels. For example, Plotnikoff et al. [54] found that intention is the mediator variable of TPB constructs for adolescents to start exercising. A number of studies suggested that multi-component interventions are more likely to succeed in developing exercise habits among youth [55,56]. It is interesting to note that, in a review by Hsu et al. [57], the most common behavior change techniques used in interventions targeting adolescents were social support, demonstration of behavior, goal setting, and feedback.

To the best of our knowledge, only a few studies have tested the effectiveness of a TPB intervention targeting only exercise behavior among youth during leisure time [58,59]. The results showed that the most important factors for the effective promotion of PA are the beliefs and the impact of information communication during the intervention. In a relevant study, Kawabata et al. [60] indicated the importance of salient beliefs in promoting PA in secondary students. In this study, Physical Education (PE) teachers were communicating persuasive messages to the intervention group during PE lessons.

Although there are numerous interventions to promote PA among youth [55], the results show a limited effect on PA levels [61,62]. It is suggested that health education programs should not isolate health behaviors, but follow an integrated approach [63]. Thus, exercise has been combined with other health-related behaviors successfully [64]. Zhang et al. [65] evaluated the effectiveness of a combined theory-based intervention on psychological effects and PA among adolescents. Their results indicated higher scores related to PA and self-efficacy in the intervention group.

### 1.3. Life Skills and School-Based Programs Targeting Healthy Diet and Exercise

Danish defined life skills as “… those skills that enable individuals to succeed in the different environments in which they live” [66]. Life skills are classified as behavioral, cognitive, social, or emotional [67], and they can lead to required abilities when they are learned and practiced successfully by an individual [68]. Additionally, life skill education has been proven more effective when implemented through a whole-school approach [69]. Young people’s risky behaviors, such as sedentarism, unhealthy diet, and time passed on media devices are worsening [70], and life skills education has been proven an effective way to promote positive behaviors and reduce unhealthy ones among school-going adolescents [71]. The literature confirms that the school setting has a key role in promoting a healthy lifestyle and preventing non-communicable diseases [72]. School education interventions are important for fostering youths’ identity development and helping them to acquire and practice skills to cope with everyday difficulties. such as problem-solving, decision-making, stress coping, and critical thinking [73]. Indeed, Hale et al. [74] reviewed multi-behavior approach programs and reported that the most effective were those that included a life skills training strategy. For example, Anand et al. [75] explored the effectiveness of an 8-month life skill school intervention promoting PA in secondary students. Skills training was distributed by experts and the results indicated a positive impact on all types of PA among students. Additionally, they stressed that health education programs that develop health-related skills and communicate knowledge and attitudes toward healthy behaviors have promising results in assisting adolescents to adopt healthy behaviors. Givaudan et al. [76] evaluated the effectiveness of the school-based program “I want to, I can… improve child nutrition and prevent diabetes”, integrating life skills education. The aim of the program was to reduce psychosocial barriers, promote PA, and prevent obesity among teachers, students, and parents. The post-intervention results reported an increase in students’ awareness of healthy eating, increased knowledge, and PA levels. Additionally, a decrease in perceived psychosocial barriers for all participants and a positive change in attitudes and social norms were observed. The literature confirms the use of life skills in effective school-based programs.

### 1.4. Aim of the Study

As several studies highlight that TPB is a well-documented framework for promoting healthy dietary behaviors [77] and PA [78], the strategies were based on the idea that students would acquire the knowledge and skills to make healthier dietary decisions and increase their PA. The hypothesis was that the students’ post-intervention measures would reflect an increase in their knowledge of healthy eating, change their behavior regarding a healthy diet, and increase their PA levels. Overall, the program comprised 10 1 h sessions, which are described below (Table 1).

## 2. Materials and Methods

### 2.1. Participants

The participants were 367 students (168 boys and 199 girls; mean age: 16.02 ± 1.19 years) from 14 secondary schools across Greece that voluntarily participated in the DIEX program. In an attempt to recruit a representative sample, we promoted the DIEX program through advertisements on social media and emails. Participants were recruited using the snowball sampling method (SSM). Fourteen teachers enrolled voluntarily in the program by fulfilling a form of participation, indicating their willingness to participate, and used the program’s materials in class according to the researcher’s guidance. Teachers informed students about the program. The inclusion criteria were being a high school student, 13–17 years old, willing to participate in the study, having parents’ consent, and being able to read and complete the questionnaires on their own. The exclusion criteria were a lack of participation in the program and an incomplete questionnaire.

### 2.2. Instruments

The following scales were used for the assessment of the DIEX health education program:

*Attitudes toward healthy eating.* Five items were used to assess attitudes toward healthy eating (e.g., “*For me taking care of my diet is…*”). The answers were provided on a 7-point bipolar Likert scale with opposing words (e.g., good–bad, silly–clever, healthy–unhealthy, pleasant–unpleasant, and useful–useless).

*Intention toward healthy eating*. This was assessed with two questions (e.g., “*I intend to take care of my diet*”). Answers were offered on a 7-point Likert scale ranging from 1 (strongly disagree) to 7 (strongly agree).

*Subjective norm.* Two items incorporated the assessment of the subjective norm, e.g., “*Important people to me believe that I will take care of my diet*”. Participants’ responses were provided on a 7-point Likert scale ranging from 1 (strongly disagree) to 7 (strongly agree).

*Perceived behavioral control*. Two items assessed students’ ability and capacity to control themselves regarding taking care of their nutrition, e.g., “*It depends on me If I take care of my diet*”. Answers were provided on a 7-point Likert scale ranging from 1 (strongly disagree) to 7 (strongly agree).

*Attitudes toward the application of the program.* Students answered one phrase “*For me, the program is …*” and provided their responses on a 7-point Likert scale with opposing words (e.g., good–bad, attractive–unattractive, useful–useless, and pleasant–unpleasant).

*Satisfaction with the program*. This was assessed by the responses to six items (e.g., “*This program* via *the internet was easy to use*”) upon the completion of the intervention and the answers were provided on a 7-point Likert scale from 1 (totally disagree) to 7 (totally agree).

*Knowledge about healthy eating*. A questionnaire was created to assess general knowledge of nutrition and diet based on previous studies by Hassandra et al. [79] and Kolovelonis et al. [80]. It consisted of 20 items (e.g., “*Junk foods are highly processed and high in calories*” or “*Poor diet and lack of exercise are linked to cardiovascular disease*”) and was delivered before and after the implementation of the educational program. Students provided their responses on a 3-point Likert scale (0 = no, 1 = I am not sure, and 3 = Yes), and the final score ranged from 0 to 60.

*Exercise behavior.* Participation in regular exercise was assessed using a single item *(“How often do you usually exercise in your free time to the extent that you get out of breath or sweat?*”). The answers were provided on a 7-point Likert scale from 0 (never) to 6 (every day) [81]. This item has already been used in studies with Greek participants [82].

All measures were completed by the students before (pre-) and/or after (post-) the implementation of the DIEX health education program. Most of the instruments used in the present study were based on the TPB [83,84]. These TPB instruments have already been translated into Greek and applied in previous studies by Theodorakis [85], Hassandra et al. [79], and Kolovelonis et al. [80].

### 2.3. Procedures

The present study examined the implementation of a school-based health education program promoting healthy diet and regular exercise in Greek secondary school students. The education program is based on the behavioral change theory, TPB, life skills education, and the use of digital elements aiming to affect the process of educating and enhancing adolescents’ lifestyles (exercise and dietary choices). The program’s sessions included behavior-modification techniques, goal setting, stress management, problem-solving, and cognitive restructuring. Teachers had online access to the educational material of the program and implemented it through class sessions. Each student completed a pre-intervention and post-intervention online questionnaire on the website of the DIEX program. Participants were recruited by social media advertisements related to the health education program. The website of the program is http://research.pe.uth.gr/nutrition/ (accessed on 28 March 2019). The distribution of the health education material through the program’s official website enabled a large sample of participants from all over Greece. Initially, teachers, who were interested in implementing the program completed a form indicating their interest in taking part in the program. In total, 14 teachers and their secondary schools from different parts of Greece responded to this call. Students and their parents also provided informed consent before participating in the program. Teachers received usernames for each student to log in and answer the pre-intervention questionnaire. The prerequisite for gaining access to the material of each program was completing the pre-intervention questionnaires. After they gained access to the educational material of the program, teachers started to implement it. The program consisted of 10 sessions of one hour each. All material was downloadable, containing application instructions, a relevant bibliography, data from research on healthy diets and PA, PowerPoint presentations, tasks, ideas for experiential activities, games, appropriate teaching strategies and techniques and guidance for practicing them, topics for project work, relevant websites, and more. Upon the completion of the program, students reused their usernames and reevaluated the programs by responding to the post-intervention questionnaires. A certificate of attendance was provided to the teachers who implemented all of the sessions and motivated their students to complete the post-intervention measurement, along with the results of the evaluation. During the implementation of the sessions, teachers were fully supported by the research team through e-mails and telephone communication.

### 2.4. Data Analysis

In each intervention, descriptive statistics (means and standard deviations) and the reliability index (Cronbach’s α) were calculated for both measurements. Then, separate paired-samples *t*-tests with Cohen’s d effect sizes were used in order to examine possible differences in the dependent variables (attitudes, intention, subjective norms, PBC, attitudes toward the program’s application, knowledge, and behavior) pre- and post-intervention programs. All statistical analyses were conducted with IBM SPSS Statistics for Windows, version 26.0 (IBM Corp, Armonk, NY, USA). The *p*-value was set at 0.05.

## 3. Results

**Figure 1:**
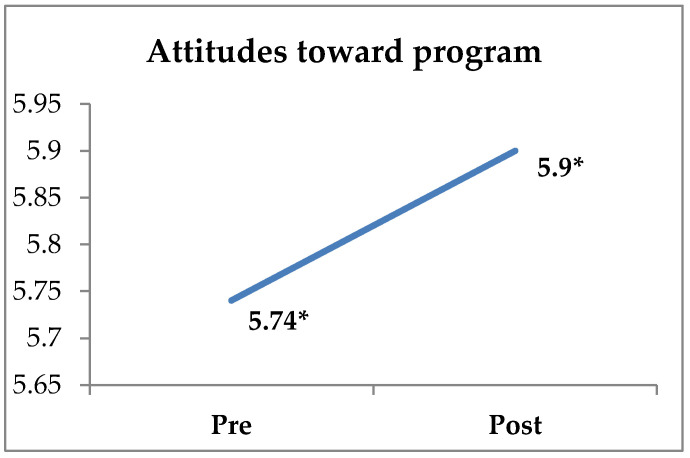
Differences between pre- and post-program measurements of attitudes toward the program’s implementation (* *p* < 0.05).

**Figure 2:**
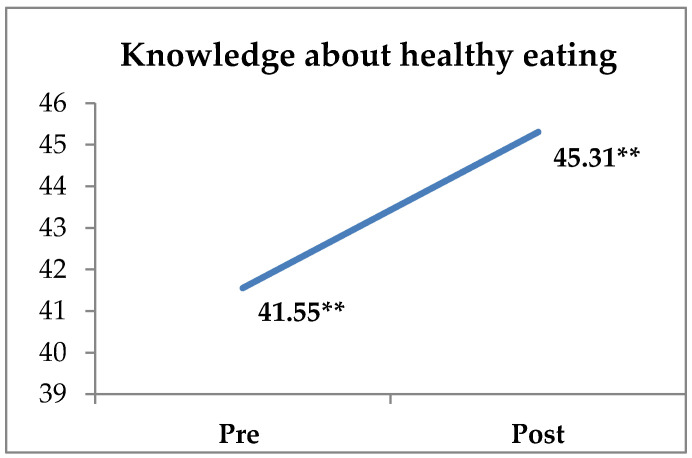
Differences between pre- and post-program measurements of knowledge about nutrition (** *p* < 0.01).

**Figure 3:**
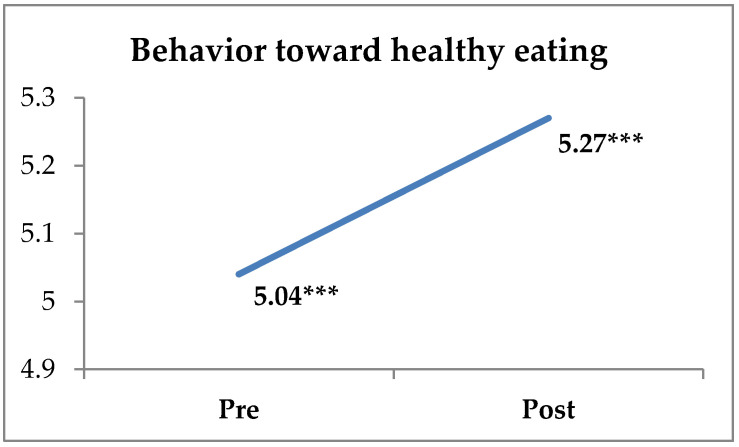
Differences between pre- and post-program measurements of nutritional behavior (*** *p* < 0.001).

The results of the descriptive statistics, reliability index, and significant differences between pre and post-intervention measurements on the DIEX health education program are presented in Table 2. More specifically, the paired-samples t-test revealed significant differences regarding students’ attitudes toward the program’s implementation (t365 = −1.996, *p* < 0.05, d = 0.10), knowledge (t365 = −6.522, *p* < 0.001, d = 0.34), and behavior toward a healthy diet (t365 = −3.182, *p* < 0.01, d = 0.17) between the pre-and post-intervention measures. Students reported higher scores for their attitudes toward the program’s application, knowledge about healthy eating, and behavior toward healthy eating after the application of the DIEX health education program compared with their pre-intervention measures (Figure 1, Figure 2 and Figure 3, Table 2). Students also reported that they were very satisfied with the application of the DIEX health education program (M = 5.83 ± 0.99). Nevertheless, there were no significant differences in the students’ attitudes toward healthy eating (t366 = −1.502, *p* = 0.134), intention toward healthy eating (t366 = 0.736, *p* = 0.462), subjective norm (t366 = −1.764, *p* = 0.079), PBC (t362 = −0.500, *p* = 0.618), and exercise behavior (t366 = 0.238, *p* = 0.812).

## 4. Discussion

The purpose of the present study was to examine the effectiveness of a school-based educational program named DIEX in promoting healthy diet and exercise to secondary school students. In general, the application of the program was satisfactory. The results indicated that the strongest effect of the program was the students’ improved knowledge (d = 0.34) about healthy eating followed by their satisfaction with the program implementation (d = 0.10). It seems that knowledge remains an easy goal to achieve by implementing such programs. Health education programs have a great impact on improving students’ knowledge, which is in accordance with previous findings [86]. The educational strategies that have been applied, such as lecturing, knowledge quizzes, and role-playing, seemed to have a great effect on students’ knowledge and their satisfaction with the program. Undoubtedly, education in specific topics has been found to increase personal awareness and skills [87]. It is interesting to note that there is a connection between knowledge about nutrition and dietary choices, but there are several factors that affect adolescents’ dietary habits [88]. Koca and Arcan, in their recent study, postulated that knowledge about healthy eating has been found to affect adolescents’ dietary habits [89]. Students reported very high scores in satisfaction with their participation in this program. These two measurements indicate that the online content of the programs satisfied students, who found it interesting, easy to access, and feasible to use. Incorporating new technologies and establishing internet school-based educational programs have been proven efficient support tools [90]. This type of intervention has the advantages of being more interactive and more attractive to adolescents [91]. In the same light, in a recent review by de Sousa et al. [92], the effectiveness of incorporating such interactive elements in programs targeting adolescents is highlighted.

On the contrary, no significant differences were found in attitudes toward healthy eating. One potential explanation of this finding may be that attitudes toward the above behavior were already positive at the pretest. Furthermore, this could reveal the difficulties in forming personal attitudes and beliefs through a short-term intervention. Similar conclusions were reached by previous research [93]. Upon further analysis, students reported significant positive attitudes toward the application of the program, demonstrating the need to incorporate such educational programs into the school curriculum.

Furthermore, their behavior toward healthy eating resulted in significantly higher post-test scores (d = 0.17). By comparing the results from Harrington et al. [38] and Arrizabalaga-López et al. [37], it seems that health education programs may positively affect participants’ behavior toward healthy eating. Moreover, no significant differences were found in the subjective norm. In the study by Evans et al. [94], adolescents perceived that their peers gave the lowest priority to healthy eating. With respect to PBC (adolescents’ perception of their control of healthy eating behavior), it is interesting to note that students reported no difference in pre- and post-program measurements. PBC may not have directly affected healthy eating, as adolescents’ choices regarding those behaviors are driven more by personal motivation than external control factors. However, this may demonstrate that adolescents feel less autonomous in what they consume, compared with adults [95], as they do not live alone and their eating choices do not depend exclusively on them, but are affected by others (e.g., parents). Contrary to this finding, Mc Dermott et al. [96], in their review, suggested that those who work with young peoples’ behaviors should emphasize the PBC factor if they want to achieve an effective intervention. Subjective norms and intentions seemed to have the weakest effect on behavior. Subjective norms (what is perceived as socially approved and desirable) typically have a weakened relationship with the intention to perform a behavior [97,98]. Regarding the exercise behavior variable, no difference between the pre- and post-test results was found. This result demonstrates that the exercise behavior rates were already high in the pre-intervention measurement [80], and the changes were minor. A possible explanation for this result could be the use of a single item to assess students’ exercise behavior.

### Limitations and Future Studies

A limitation of the present study was that students’ behaviors were assessed by self-reported questionnaires. Thus, their answers might be affected by recall bias. In future studies, the assessment could be based on teachers’ or parents’ proxy self-reporting instruments, if possible, as parental involvement is suggested for more effective healthy diet educational interventions [96]. It is also vital to mention that the implementation of the present program and the results of the evaluation may contribute to the personal and professional development of the participating teachers or possible future ones. It is advisable for a teacher to be well-educated and informed on health education programs to implement them effectively [97]. The absence of a control group should also be noted, as this limitation may lead to unsafe conclusions regarding the causal effect of the present intervention treatment. Despite the aforementioned limitations, although effectiveness studies, such as the present one, may sacrifice some internal validity, they have higher external validity than efficacy studies. The strengths of the current study were the combination of TPB components, life skills education, the use of technology, the use of theory-based questionnaires for data collection, and the use of a supportive booklet during the sessions’ delivery.

## 5. Conclusions

Considering the results of this study, the most prominent evidence from the application of this educational school program is the knowledge factor. Furthermore, it was successful in changing attitudes toward the program and behavior toward a healthy diet, but not for subjective norms, perceived behavioral control, and intentions. In addition, students reported high scores in satisfaction with the program’s application. These findings have great implications for healthy diet promotion interventions among young people, especially in Greek schools. A theory-driven and skill-improvement educational intervention was effective among adolescents.

## Figures and Tables

**Table 1:** Description of the DIEX program (sessions and activities).

Sessions	Contents	Activities
1st	Introduction of the program—Purpose of the program— Determine way of working—Initial evaluation	Students grouped—I learn about my body—What is BMI (Body Mass Index)?—What are metabolism and calories?—Students calculate their BMI—Communicate with significant others
2nd	What is a healthy diet—Macronutrients—Table with nutrient information—Food pyramid	Knowledge test of healthy diet—Create a food pyramid poster with a collage of images of foods—Learn to read food labels—Create a message about nutritive food
3rd	Lead causes of unhealthy diet—Consequences of poor diet—Eating disorders and their causes	Knowledge test of unhealthy foods—Analyze campaigns promoting the ideal body type—Create a message reflecting this session and communicate it to others.
4th	Obesity—Data from Hellenic Medical Society of Obesity—Physical and mental health effects of obesity—Childhood obesity data—The psychology of obesity	Demystification of the ideal body—Teamwork: “To what extent do we judge ourselves and others by our/their appearance”
5th	Diet—Restrictions on a diet—How mass media promotes unhealthy food products as healthy choices—Hidden messages—The effectiveness of a diet is related to psychology—The Forbidden Fruit Theory—How to monitor our eating habits	Personal assessment questionnaire—Interviewing a family member who makes effort to lose weight with no results—Keep a food diary—Create a message of the session
6th	Regular exercise and its importance—Research data of PA benefits in physical and psychological health—List of calories burned in 1 h of exercise	Knowledge test of the health benefits of exercise—Self-assessment questionnaire: “Why do I not exercise?”—Keep a PA diary—“Creating the curve of monthly PA”
7th	Changing our diet and PA habits—Reflection on our healthy and unhealthy behaviors—Assessment of eating behavior	Teamwork: “Create a balanced diet plan”—Complete a personal diet and PA diary—Step-by-step guidance for the goal-setting technique (Exercise behavior)—Create and communicate the message of the session
8th	Models of behavior change—Controlling our emotions—Introducing control strategies	Strategies for developing emotional intelligence—Regulate our emotions—Positive self-talk—Awareness—Role-playing—Controlling thoughts—Relaxation techniques—Modifying outcome expectations
9th	Setting goals for a healthy diet and regular exercise—Introduce goal-setting theory—What type of goals should I set?—Individual and team practice with the goal-setting skills for fitness issues	Step-by-step goal-setting technique (healthy diet behavior)—Overcoming obstacles to health (healthy dietary and exercise behavior)—Create the message of the session and communicate it to significant others
10th	Publication of the program to other students, teachers, parents, and others—Learn how to transfer knowledge to significant others	Organize a lecture by health experts (psychologists, nutritionists, and fitness trainers) and obtain information about health issues—Organize a celebration with a buffet of healthy foods using all of the information gathered—Exhibition of the material of the program (posters, photos, images, and messages)—Presentation

**Table 2:** Descriptive statistics, reliability index, and significant differences between pre- and post-intervention measures for the DIEX health education program.

	Pre-Intervention			Post-Intervention		
Variables	α	M	SD	α	M	SD
Attitudes toward healthy eating	0.80	6.06	0.90	0.81	6.14	0.94
Intention toward healthy eating	0.77	6.22	0.98	0.90	6.17	1.16
Subjective norm	0.81	5.85	1.22	0.87	5.98	1.22
PBC	0.44	6.18	0.94	0.64	6.21	0.98
Attitudes toward the program *	0.82	5.74	1.02	0.56	5.90	1.48
Knowledge about healthy eating ***	0.74	41.55	9.21	0.79	45.31	11.26
Behavior about healthy eating **	0.81	5.04	1.30	0.88	5.27	1.40
Exercise behavior	-	4.59	1.17	-	4.57	1.27
Satisfaction with the program	-	-	-	0.84	5.83	0.99

*Note:* M: Mean; SD: standard deviation; PBC: perceived behavioral control; α: Cronbach’s alpha reliability index; * *p* < 0.05; ** *p* < 0.01; *** *p* < 0.001.

## Data Availability

The data underlying the results presented in the study are part of a research program and are available on request to the corresponding author (Y.T.; theodorakis@uth.gr).
